# XocR, a LuxR solo required for virulence in *Xanthomonas oryzae* pv. *oryzicola*

**DOI:** 10.3389/fcimb.2015.00037

**Published:** 2015-04-16

**Authors:** Huiyong Xu, Yancun Zhao, Guoliang Qian, Fengquan Liu

**Affiliations:** ^1^Institute of Plant Protection, Jiangsu Academy of Agricultural ScienceNanjing, China; ^2^College of Plant Protection, Nanjing Agricultural UniversityNanjing, China; ^3^Key Laboratory of Integrated Management of Crop Diseases and Pests (Nanjing Agricultural University), Ministry of EducationNanjing, China

**Keywords:** *Xoc*, QS, LuxR, virulence, motility

## Abstract

*Xanthomonas oryzae* pv. *oryzicola* (*Xoc*) causes bacterial leaf streak (BLS) in rice, a serious bacterial disease of rice in Asia and parts of Africa. The virulence mechanisms of *Xoc* are not entirely clear and control measures for BLS are poorly developed. The solo LuxR proteins are widespread and shown to be involved in virulence in some plant associated bacteria (PAB). Here, we have cloned and characterized a PAB LuxR solo from *Xoc*, named as XocR. Mutation of *xocR* almost completely impaired the virulence ability of *Xoc* on host rice, but did not alter the ability to trigger HR (hypersensitive response, a programmed cell death) on non-host (plant) tobacco, suggesting the diversity of function of *xocR* in host and non-host plants. We also provide evidence to show that *xocR* is involved in the regulation of growth-independent cell motility in response to a yet-to-be-identified rice signal, as mutation of *xocR* impaired cell swimming motility of wild-type Rs105 in the presence but not absence of rice macerate. We further found that *xocR* regulated the transcription of two characterized virulence-associated genes (*recN* and *trpE*) in the presence of rice macerate. The promoter regions of *recN* and *trpE* possessed a potential binding motif (an imperfect *pip* box-like element) of XocR, raising the possibility that XocR might directly bind the promoter regions of these two genes to regulate their transcriptional activity. Our studies add a new member of PAB LuxR solos and also provide new insights into the role of PAB LuxR solo in the virulence of *Xanthomonas* species.

## Introduction

Quorum sensing (QS) is a cell-density dependent cell-cell communication system that relies on small chemical signal molecules to control bacterial behavior and coordinate gene expression in a cell-density dependent manner (Fuqua et al., 1994). The QS-controlled bacterial behaviors are diverse, including biofilm formation, cell motility, antibiotic production, light production and sporulation (Nealson et al., 1970; Danhorn and Fuqua, 2007; Goo et al., 2010; Qian et al., 2014). In Gram-negative bacteria, the *N*-acyl homoserine lactones (AHLs) are a major class of signal molecules. This signal is composed of a homoserine lactone ring carrying C_4_-C_18_ acyl chains and is thought to function as intraspecies communication (Fuqua et al., 2001; Fuqua and Greenberg, 2002; Ng and Bassler, 2009).

A typical AHL QS system is composed of homologs of the LuxI and LuxR proteins that were first identified in *Vibrio fischeri* (Nealson et al., 1970; Fuqua and Winans, 1996). LuxI homologsare AHL synthases and direct the synthesis of autoinducer molecules. A LuxR-family protein that is part of a QS system has a *N*-terminal AHL-binding and a C-terminal DNA-binding domains. These LuxR-family proteins can bind AHLs, and consequently, the stable LuxR-AHL complex binds at specific gene regulatory sequences called as *lux* box to activate or repress the transcription of target genes (Fuqua et al., 2001). Notably, these two proteins are in most cases genetically adjacent to each other in a canonical AHL QS system.

Additional LuxR-family proteins similar to QS LuxR homologs are being identified by analysis of sequenced bacterial genomes and many of these are without a cognate LuxI protein. These LuxR proteins lacking a genetically linked LuxI have been termed LuxR orphans or solos (Fuqua, 2006; Patankar and Gonzalez, 2009; Subramoni and Venturi, 2009; Gonzalez et al., 2013). Studies so far show that some LuxR solos respond to endogenous AHLs (e.g., QscR from *Pseudomonas aeruginosa*) or to exogenous AHLs produced by neighboring bacteria, such as the SdiA from *Salmonella enterica* and *E. coli* to regulate target genes (Ahmer, 2004; Fuqua, 2006).

Recently, a sub-group of LuxR solos has been identified to be very common in several plant-associated bacteria (PAB); these PAB LuxR solos do not bind AHLs, but respond to yet unidentified plant signal(s)/compound(s) (Ferluga et al., 2007; Chatnaparat et al., 2012). PAB LuxR solos differ in the conservation of one or two of the six invariant amino acids in the AHL-binding domain that have been reported to be important for signal/ligand binding (Gonzalez and Venturi, 2013). Examples of this subfamily are required for full virulence in several phytopathogenic bacteria and include XccR of *Xanthomonas campestris* (Zhang et al., 2007); OryR of *X. oryzae* (Ferluga et al., 2007); XagR of *X. axonopodis* (Chatnaparat et al., 2012) and PsaR2 of *P. syringae* pv. *actinidiae* (Patel et al., 2014).

Bacterial leaf streak (BLS) caused by *X. oryzae* pv. *oryzicola* (*Xoc*) is an important disease of rice in Asia and parts of Africa (Nino-Liu et al., 2006). *Xoc* is a Gram-negative bacterium which produces a characteristic yellow pigment. This pathogen penetrates the rice leaf mainly through stomata, multiplies in the substomatal cavity, and colonizes the parenchyma apoplast causing interveinal lesions (Wang et al., 2007). *Xoc* can also gain access through wounds, but does not invade the xylem because it is restricted by the mesophyll tissue apoplast (Nino-Liu et al., 2006). We identified a PAB LuxR solo homolog in the genome of *Xoc* strain BLS256 and designated it as XocR. In the present study, the role of *xocR* in virulence as well as cell motility is presented and discussed.

## Materials and methods

### Bacterial strains, culture media, and growth conditions

The bacterial strains and plasmids used in this study are listed in Table S1. *Xoc* strains were grown at 28°C in nutrient broth (NB) medium (beef extract, 3 g/l; yeast extract, 1 g/l; polypeptone, 5 g/l; sucrose, 10 g/l) or on nutrient agar (NA). Medium containing macerated rice material was prepared as described previously (Gonzalez et al., 2013). *Escherichia coli* strains were cultivated at 37°C in Luria-Bertani (LB) medium or on LB agar plates. When required, antibiotics were added to the medium at the following final concentrations: 50 μg/mL kanamycin (Km) for *E. coli* and *Xoc*, 10 μg/mL gentamicin (Gm) for *E. coli* and *Xoc*, and 100 μg/mL rifampicin (Rif) for *Xoc*.

### Generation of the XocR deletion mutant in X. oryzae Pv. oryzicola

Deletion mutants were generated as described previously (Qian et al., 2013b). The *Xoc* wild-type Rs105 was used as the parental strain to generate the in-frame deletion mutant via allelic homologous recombination. In-frame deletion of *xocR* was performed as described previously (Figure S1A). Briefly, two *xocR* flanking regions were generated by polymerase chain reaction (PCR) using the primer pairs *xocR*-1F/*xocR*-1R and *xocR*-2F/*xocR*-2R (Table S2). The *xocR*-1 fragment (digested with *Bam*HI and *Hin*dIII) and the *xocR*-2 fragment (digested with *Hin*dIII and *Xba*I) were ligated into *Bam*HI/*Xba*I-digested pK18mob*sacB* (Schafer et al., 1994). This construct, designated pK18-xocR, was transformed into the wild-type Rs105 by electroporation. Transconjugants were selected on NA plates without sucrose but with Rif (100 μg/mL) and Km (50 μg/mL). Positive colonies were plated on NA plates containing 10% (w/v) sucrose and Rif (100 μg/mL) to select for resolution of the construct by a second cross-over event. The resulting mutant, containing the *xocR* in-frame deletion, was confirmed by PCR (Figure S1B). One of the confirmed mutants, named as Δ*xocR*, was selected for further study.

### Complementation of the XocR mutant

The generation of complemented strains was performed as described previously (Qian et al., 2013a). Briefly, a 1664-bp DNA fragment containing *xocR* and its predicted promoter region was amplified from wild-type Rs105 with xocRH-F/xocRH-R primers (Table S2). The PCR fragment was digested with the *Bam*HI/*Xba*I enzyme and cloned into *Bam*HI/*Xba*I-digested pBBR-MCS5 (Kovach et al., 1995) resulting in the complemented construct (pBBR-XocR). This construct was transformed into Δ*xocR* competent cells by electroporation. Finally, one positive complemented strain named Δ*xocR*(*xocR*) was selected on NA plates with Rif and Gm, and verified by PCR method. This final complemented strain, named as Δ*xocR* (*xocR*) was used for further study.

### Pathogenicity testing and determination of bacterial load *in planta*

The pathogenicity testing and determination of bacterial load assays were performed as described previously (Guo et al., 2012; Qian et al., 2013b). Briefly, *Xoc* strains were incubated in NB broth with appropriate antibiotics at 28°C until the growth reached to exponential phase (OD_600_ = 0.5). Then, cells were pelleted by centrifugation at 3099 × g and suspended in an equal volume of sterilized ddH_2_O. The final cell suspension was inoculated into the leaves of 2-week-old rice plants (Shanyou-63, susceptible to BLS) using needless syringe. Water-soaking symptoms were measured 7 days after inoculation. Twenty-five leaves were inoculated for each *Xoc* strain in each treatment. The same experiment was performed three times.

Growth of each *Xoc* strain in rice leaf tissue was detected by homogenizing five inoculated leaves in 9-mL sterile water. The leaves were cut in 6-mm sections around the inoculation spots on days 0, 7 after inoculation (Lee et al., 2008). Diluted homogenates were plated on NA plates supplemented with Rif (for the wild type and mutant). The number of bacterial colonies on these plates was counted after 2 days of incubation at 28°C (Feng et al., 2009). Each diluted homogenate was plated on three plates, respectively. Three replicates for each treatment were used, and the experiment was performed three times.

### Hypersensitive response (HR) assay

The hypersensitive response assay was performed as described previously (Zou et al., 2006; Qian et al., 2013b). *Xoc* strains were incubated in NB broth with appropriate antibiotics at 28°C until exponential phase (OD_600_ = 0.5). Cells were pelleted by centrifugation at 3099 × g and suspended in different volume of sterilized ddH_2_O resulting at three different concentrations of cell suspension (OD_600_ = 0.2, 0.4, and 0.8). These three cell suspensions were used for HR tests. Briefly, *Xoc* strains were infiltrated into the leaves of greenhouse-grown tobacco (*Nicotiana tabacum* L. cv. *Samsun*), and the results were observed after 48 hours of infiltration. Four leaves were inoculated for each *Xoc* strain in each treatment. The same experiment was performed three times.

### Determination of bacterial growth ability *in vitro*

Bacterial growth was monitored as described previously (Qian et al., 2013b). To investigate bacterial growth *in vitro*, we tested the growth rate of Δ*xocR* in nutrient-rich broth (NB) and NB with rice macerate medium. In brief, *Xoc* strains were pre-incubated in NB broth at 28°C with shaking at 200 rpm, until the growth was reached to OD_600_ of 1.0. Cells were pelleted by centrifugation at 3099 × g and suspended in an equal volume of sterilized water. Then, 1 mL of cell suspension was inoculated into 100 mL of two testing media. All inoculation broths were grown at 28°C with shaking at 200 rpm and the OD_600_value was determined every 4 h until bacterial growth reached to the stationary stage. The experiments were performed three times, and each involves three replicates.

### Determination of cell motility, EPS production, protease activity, and biofilm formation between wild-type strain and the XocR mutant in the absence or presence of rice macerate

Swimming motility assay was performed as described previously (Gonzalez et al., 2013). In brief, *Xoc* strains were grown in nutrient-rich broth (NB) broth at 28°C with shaking at 200 rpm, until the value of OD_600_ reached 1.0, and then 3 μl of each strain was inoculated onto the surface of the motility plates, including NA containing 0.3% soft agar with and without of rice macerate. Cell motility was examined at 72 h after incubation at 28°C. Five independent motility assays were performed for each strain.

EPS production of the bacterium was measured as described previously with some modifications (Tang et al., 1991). Briefly, *Xoc* strains were pre-incubated in NB broth at 28°C with shaking at 200 rpm, until the value of OD_600_ to be 1.0. Cells were pelleted by centrifugation at 3099 × g and suspended in an equal volume of sterilized ddH_2_O. Then, 0.5 mL of cell suspension was inoculated into 50 mL of NB or NB with rice macerate. These cultures were grown at 28°C with shaking at 200 rpm for 5 days. EPS was precipitated from the culture supernatant with two volumes of ethanol and dried to a constant weight at 80°C. The difference between the two weights was used to estimate the production of EPS per milliliter of culture. Each experiment was performed three times, and each treatment involved three replicates.

To measure extracellular protease activity, *Xoc* strains were incubated in NB medium at 28°C with shaking at 200 rpm, until the OD_600_ to be 1.0. Then, 3 μL of bacterial culture was spotted on NA plates or NA with rice macerate. Both media contained 1% (m/v) skim milk powder. After 48 h of incubation at 28°C, protease activity was assessed according to the hydrolytic zones around the bacterial colonies (Ryan et al., 2006). Each treatment involves three replications, and the same experiment was performed three times.

To test biofilm formation on abiotic surfaces, *Xoc* strains were pre-incubated in NB broth at 28°C with shaking at 200 rpm, until the value of OD_600_ to be 1.0. Cells were pelleted by centrifugation at 3099 × g and suspended in an equal volume of sterilized ddH_2_O. Then, 30 μL of cell suspension was inoculated into 3 mL of NB or NB with rice macerate. These cultures were incubated at 28°C without shaking for 5 days. After gentle removing the suspension, two volume of 10% crystal violet solution was added and treated for 1 h, then the glass tubes were gently washed three times with sterile ddH_2_O and air dried for 1 h; then, 3 mL of 40% methanol and 10% glacial acetic acid was added to glass tubes to dissolve the crystal violet stain (Koczan et al., 2009). The dissolved crystal violet was quantified through spectrophotometry at an absorbance of 575 nm by using a Safire microplate reader (Tecan, Research Triangle Park, NC). Each experiment included three replicates and experiments were repeated three times.

### Real-time PCR

The assays of RNA extraction, cDNA synthesis and real-time PCR were performed as described previously (Chatnaparat et al., 2012; Qian et al., 2013b). Quantitative real-time reverse transcription PCR (qRT-PCR) was carried out using a SYBR Premix EX Tag™ II kit (TaKaRa Bio, Shiga, Japan) in an ABI PRISM 7500 Real-Time PCR System (FApplied Biosystems, Foster City, CA, USA). As the endogenous control, 16S rRNA was used. The primer sequences were listed in Table S3. RNA was extracted from *Xoc* wild-type strain Rs105 and Δ*xocR* by using the RNAiso Plus Reagent (TaKaRa Bio) following the manufacturer's instructions. To remove genomic DNA, the eluted RNA samples were treated with RNase inhibitors and DNaseI (TaKaRa Bio). RNA integrity was confirmed by electrophoresis on 1.2% agarose gels. Then, 2 μg of each RNA sample was used to synthesize cDNA with a cDNA Synthesis kit (TaKaRa). The same experiment was performed three times. The transcriptional levels of *XOC_1737* and *XOC_4211* in the wild-type Rs105 and the *xocR*-deletion mutant were also assessed and compared.

## Results and discussion

### XocR of *Xoc* belongs to a sub-family of PAB LuxR solos

**Figure 1:**
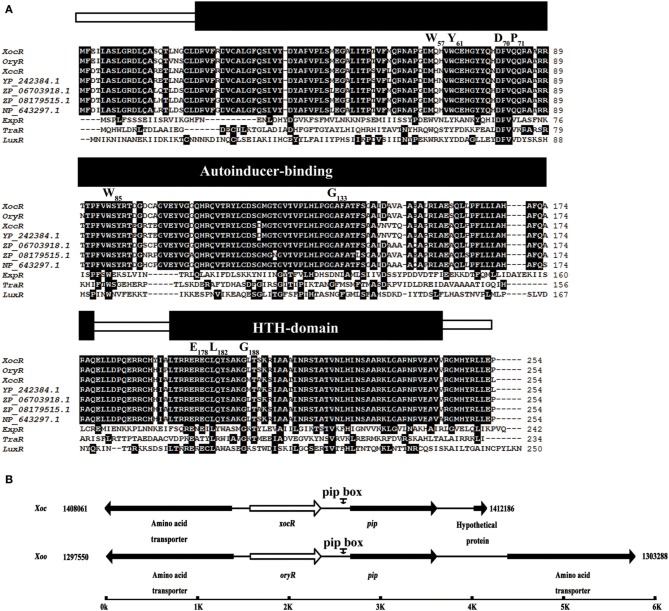
**Sequence characterization of XocR in *Xanthomonas oryzae* pv. *oryzicola*. (A)** Alignment analysis of LuxR-type transcriptional regulators. Exception to XocR and ExpR, other solo LuxRs used in this figure are described previously (Gonzalez and Venturi, 2013), and they are: OryR, *Xanthomonas oryzae* pv. *oryzae* KACC10331; XccR, *X. campestris* pv. *campestris* 8004; YP_242384.1, *X. gardneri* ATCC 19865; ZP_06703918.1, *X. fuscans* subsp. *aurantifolii* ICPB 11122; ZP_08179515.1, *X. vesicatoria* ATCC 35937; NP_643297.1 *X. axonopodis* pv. *citri* 306; QS LuxR: ExpR of *Pectobacterium carotovorum* subsp. *carotovorum*; TraR of *Agrobacterium tumefaciens*; LuxR of *Vibrio fischeri*. The nine highly conserved amino acids are highlighted in black and their positions with respect to TraR are indicated above. **(B)** Comparison of *xocR* and *oryR* organization in the genome of *X*. *oryzae* pv. *oryzicola* BLS256 and *X*. *oryzae* pv. *oryzae* KACC10331, respectively. *xocR* and *oryR* were both highlighted in white.

To examine whether the genome of *Xoc* has potential LuxR solos, OryR which is a PAB LuxR solo from *Xanthomonas oryzae* pv. *oryzae* (*Xoo*; which is a very closely species to *Xoc*), was selected as a subject to perform a BlastP search in the genome of *Xoc* strain BLS256 (Accession number: NC_017267). This led to the identification of a corresponding ortholog, termed as XocR (XOC_1422), which shared 97% similarity/identity with OryR in sequence at amino-acid level. To determine whether XocR is a member of the PAB LuxR solos, the sequence features of XocR and its genomic organization in *Xoc* were further analyzed. XocR possessed a typical AHL-binding domain at the N terminus and a DNA-binding HTH domain at the C terminus (Figure 1). Furthermore, *Xoc* genome did not have a cognate *luxI* and hence XocR is a LuxR solo in *Xoc*. Importantly, as like other PAB LuxR solos, XocR had an imperfect AHL-binding domain with substitutions two of the highly conserved amino acids in the AHL binding domain (Covaceuszach et al., 2013; Gonzalez and Venturi, 2013). More specifically, W57 and Y61, the amino acid with the number in respect to TraR, a canonical QS LuxR protein in *Agrobacterium tumefaciens* (Covaceuszach et al., 2013; Gonzalez and Venturi, 2013), were substituted by methionine (M) and tryptophan (W), respectively (Figure 1A). Collectively, these results indicated that XocR belongs to the sub-family of PAB LuxR solos, which binds and responds to the yet-to-be-identified plant-derived compound(s). Further analysis was carried out to identify additional four genes that were genetically adjacent to *xocR* in the genome of *Xoc* strain BLS256. Specifically, *XOC_1420* and *XOC_1421*, which encodes a hypothetical protein and an amino acid transporter, respectively, were located closely in the upstream of *xocR* (Figure 1B). This organization is similar to gene arrangement in *Xoo* (Figure 1B). However, we also found a difference in the nucleotide sequence of two genes located downstream of *xocR* compared to those of *Xoo*. The observed difference between *Xoo* and *Xoc* was the presence of a gene (*XOC_1424*) encoding a hypothetical protein located closely to the *pip* gene in *Xoc*, which was absent in *Xoo*. In *Xoo* on the other hand at the similar position, a locus (*XOO1270*) encoding a protein that putatively functions as an amino acid transporter was located adjacent to the *pip* gene (Figure 1B). Our results suggest that XocR is a member of a sub-family of PAB LuxR solos. Notably, although *Xoo* and *Xoc* are two pathovars of *X. oryzae*, XocR of *Xoc* also exhibited distinctive features in sequence (e.g., sequence organization) with that of well-characterized OryR from *Xoo*. These findings prompted us to test the potential role of *xocR* in the virulence of *Xoc* as well as other important pathogenesis related traits (see below).

### XocR is required for *Xoc* virulence in host rice, but is not necessary for trigging HR in non-host (plant) tobacco

**Figure 2:**
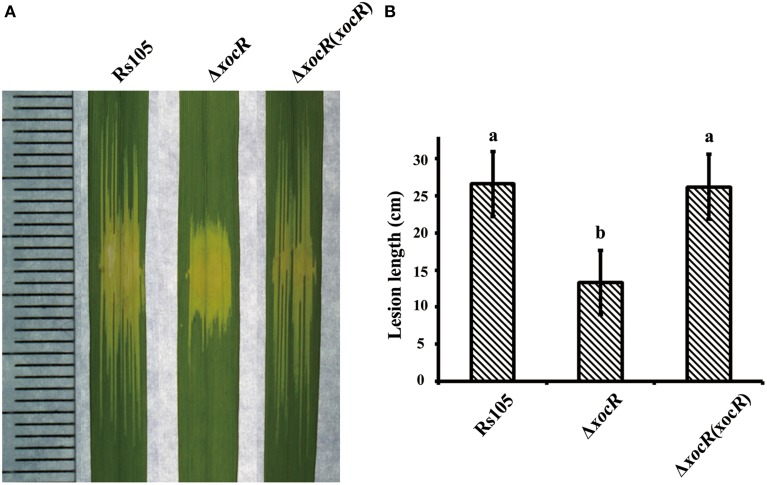
**Mutation of *xocR* impaired the virulence of *Xanthomonas oryzae* pv. *oryzicola*. (A)** Representative result of water-soaking lesion lengths on the rice seedling leaves (cv. Shanyou63, 2-week old) by infiltration with wild-type Rs105 and the *xocR* mutant. **(B)** Calculated data of water-soaking lesion lengths on the leaves of rice seedling leaves. Rs105, wild-type strain; Δ*xocR*, the *xocR* deletion mutant; Δ*xocR* (*xocR*), the complemented strain of the *xocR* deletion mutant. Three replicates were used for each treatment, and the experiment was performed three times. Vertical bars represent standard errors. Different letters above data bars indicate a significant difference between the wild-type strain and tested mutants (*P* < 0.05; *t*-test).

In order to gain insight into the role of *xocR* in *Xoc* virulence, we generated the *xocR* in-frame deletion mutant as well as the corresponding complemented strain (Figure S1). Pathogenicity tests showed that mutation of *xocR* almost completely impaired in virulence, whereas the complemented strain regained virulence to wild-type levels (Figures 2A,B). This finding suggested that *xocR* was required for *Xoc* virulence on host rice. We were then interested to determine whether *xocR* was also required for triggering the HR (a programmed cell death) in non-host (plant) tobacco. For this purpose, three different cell concentrations of wild-type Rs105 and the *xocR* mutant were infiltrated into the leaves of tobacco. We determined that mutation of *xocR* did not alter the ability to trigger HR in tobacco under all testing conditions and behaved as the wild-type (Figure S2). This point indicated that *xocR* was not involved in trigging HR in non-host (plant) tobacco. Our results also provide a clue to reveal the diversity of function of *xocR* in host and non-host plants. The PAB LuxR solos have been shown to mediate virulence in diverse phytopathogenic bacteria by binding yet-to-be-identified plant signal/compound; these are XccR of *X. campestris* binding a signal present in cabbage (Zhang et al., 2007), OryR of *X. oryzae* binding a signal compound present in rice (Ferluga and Venturi, 2009) and XagR of *X. axonopodis* binding a signal molecules in soybean, rice and cabbage but not in tobacco (Chatnaparat et al., 2012). These previous observations raised the possibility that binding a signal/compound in rice leaves, but not in tobacco by XocR probably contributed to probable roles of XocR in host rice and non-host tobacco.

### Mutation of XocR does not affect the growth ability *in planta* and *in vitro*

The finding of virulence deficiency of the *xocR* mutant prompted us to examine whether this trait is associated with the *in planta* growth capacity of the mutant. To test this, we recovered the bacterial cells from the infected rice leaves after inoculation of 7 days. As shown in Figure S3, a mutation in *xocR* did not alter the bacterial cell populations when compared to the ones of the wild-type strain Rs105 *in planta*, as the bacterial numbers recovered from infected rice leaves between wild type and the *xocR* mutant were similar under our testing conditions. To further study growth ability, the *in vitro* growth profiles in different media of wild-type Rs105 and the *xocR* mutant were determined. As shown in Figure S4, it was clearly observed that the *xocR* mutant always displayed wild-type growth ability either in nutrient-rich medium or NB with rice macerate medium. Collectively, these results indicate that the virulence deficiency of the *xocR* mutant on host rice was probably not due to the *in planta* growth impairment. However, our result is not consistent with the finding on the role of PsaR2 in plant colonization, which is another PAB LuxR solos from *P. syringae* pv. *actinidiae*, a pathogen of kiwifruit (Patel et al., 2014). In this bacterium, mutation of *psaR2* impaired the ability of *in planta* colonization and virulence. These findings therefore suggest that PAB LuxR solos may play different roles in plant colonization in different phytopathogens.

### XocR is involved in the regulation of cell swimming motility in the presence of rice signal

**Figure 3:**
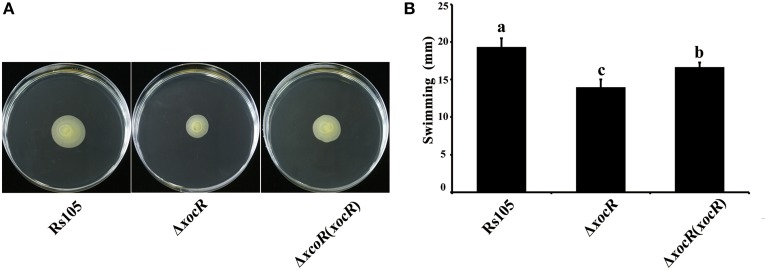
**Mutation of *xocR* impaired cell swimming motility of *Xanthomonas oryzae* pv. *oryzicola* in the presence of rice macerate. (A)** Representative result of motility haloes of wild-type Rs105 and the *xocR* deletion mutant; **(B)** Calculated data of diameters of the swimming haloes on the plate of NA with rice macerate by tested *Xoc* strains. Rs105, wild-type strain of *X*. *oryzae* pv. *oryzicola*; Δ*xocR, xocR*-deletion mutant of *X*. *oryzae* pv. *oryzicola*; Δ*xocR* (*xocR*), the complemented strain of the *xocR* deletion mutant. Three replicates were used for each treatment, and the experiment was performed three times. Vertical bars represent standard errors. Different letters above data bars indicate a significant difference between the wild-type strain and tested mutants (*P* < 0.05; *t*-test).

The finding that virulence deficiency of the *xocR* mutant was not associated with its growth impairment *in planta*, suggested that XocR may utilize a novel mechanism(s) to regulate virulence in *Xoc*. This prompted us to examine whether any of the four well-studied virulence-associated traits, including cell motility, EPS production, biofilm formation and extracellular protease activity were impaired in the *xocR* mutant, resulting in virulence deficiency. We therefore determined the ability of the *xocR* mutant in regulating these phenotypes. As shown in Figure S5, we did not find any visible difference in the tested phenotypes between wild-type Rs105 and the *xocR* mutant in the absence of rice macerate. As reported previously, the regulation of the PAB LuxR solos with regard to phenotypes occurs to the greatest extent in response to the presence of plant signal(s) (Chatnaparat et al., 2012; Gonzalez et al., 2013). To test this possibility, we re-examined these four tested virulence-associated traits between wild-type Rs105 and the *xocR* mutant in the presence of rice macerate. Using this procedure, we observed that *xocR* was involved in the regulation of cell motility and there was no difference in the other three phenotypes (EPS production, biofilm formation and extracellualr protease) (Figure S6). As shown in Figure 3, we observed that a mutation in *xocR* significantly reduced the cell swimming motility of wild-type strain in rich medium supplemented with rice macerate, whereas the cell swimming motility of the *xocR* complemented strain was found to be partially restored to the wild-type level under the same testing conditions (Figure 3). Furthermore, we also determined that the *xocR* mutant displayed wild-type growth level in this medium (Figure S4B), suggesting the regulation of *xocR* in cell swimming motility in the presence of rice macerate was not due to any difference in the growth of *Xoc* derivatives. As cell swimming motility can be associated with bacterial growth and EPS production in diverse bacteria (Ali et al., 2000), we tested these phenotypes and determined that the regulation by *xocR* of cell swimming motility was not linked to these two phenotypes in *Xoc* since a mutation of *xocR* did not impair these two traits both in the presence or absence of rice signal(s). Similarly to what occurs in *Xoc*, cell swimming motility is also regulated by OryR in *Xoo* (Gonzalez et al., 2013).

In *Xoo*, OryR was found to positively control the transcriptional expression of a number of movement-related genes, including 30 flagella genes in response to rice macerate (Gonzalez et al., 2013). Further studies showed that OryR directly regulated most probably via a *pip* box-like sequence of the promoter region of *flhF*, the flagella-regulator-encoding gene (Gonzalez et al., 2013). A BlastP search in *Xoc* demonstrated a similar organization of these flagellar genes in *Xoc* (Table S5). This finding raised the possibility that XocR might use pathway/mechanism similar to OryR to modulate cell motility in *Xoc*. It must be noted that *Xoo* is a vascular pathogen unlike *Xoc*, raising the question whether OryR and XocR respond to the same rice signal/compound(s). Identification of the rice signal(s) that interacts with OryR or XocR will give more insight into this point. Nevertheless, our results suggest that response to host signal is important for XocR to regulate cell motility, which seems to facilitate the infection of *Xoc* on host rice.

### XocR controls the transcription of two known virulence-associated genes in the presence of rice signal

**Figure 4:**
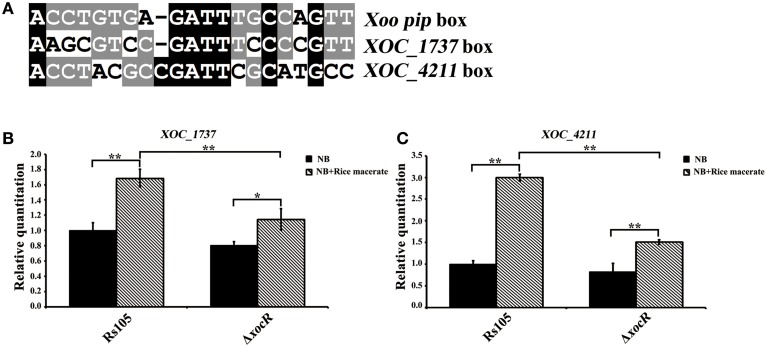
**The contribution of rice macerate to the regulation of *xocR* on the transcription of two known virulence-associated genesn *Xanthomonas oryzae* pv. *oryzicola*. (A)** Sequence alignment of the characterized *Xoo pip* box and the promoter regions of two characterized virulence-associated genes (*XOC_4211* and *XOC_1737*) in *Xoc*. **(B,C)** Determination of the transcriptional level of *XOC_1737* and *XOC_4211* between wild-type Rs105 and the *xocR* deletion mutant (Δ*xocR*), respectively in the absence or presence of rice macerate. *XOC_1737* encodes a DNA repair protein (RecN) and *XOC_4211* encodes an anthranilate synthase component I (TrpE). Three replicates were used for each treatment, and the experiment was performed three times. Vertical bars represent standard errors. The data was analyzed by One-Way ANOVA. Tukey HSD *post-hoc* comparisons were reported, where ^**^P < 0.001, ^*^P < 0.005.

In addition to cell motility, we were interested to examine whether XocR regulates other regulatory mechanism(s) to mediate virulence. As described above, XocR most likely binds to a DNA sequence called *pip*-box present in the promoter region of target genes to regulate their transcription. In view of this, an analysis of the promoter sequences of 29 reported virulence-associated genes of *Xoc* was made (Table S4); this led to the discovery of two candidate genes that might be regulated by XocR at transcriptional level, since their promoter regions possessed a potential binding motif (an imperfect *pip* box element) (Figure 4A). They are *XOC_1737*, encoding a DNA repair protein (RecN) and *XOC_4211*, encoding an anthranilate synthase component I (TrpE). To address whether the transcription of these two genes was controlled by *xocR*, we used qRT-PCR to determine their transcriptional levels in wild-type Rs105 and the *xocR* mutant. As shown in Figures 4B,C, compared to control medium (NB), addition of rice macerate significantly induced the transcriptional expression of *recN* (~1.7-fold) and *trpE* (~3.0-fold) in the wild-type background. However, the induced transcriptional effect of *recN* and *trpE* by rice macerate was reduced to ~1.4 and ~1.8-fold in the *xocR* mutant, respectively, compared to the control medium. Meanwhile, the transcription of *recN* or *trpE* in the wild-type strain and the *xocR* mutant was similar in the absence of rice macerate, whereas in the presence of rice macerate, mission of *xocR* significantly alter the expression pattern of both genes. In details, the expression level of *recN* and *trpE* was reduced to (~1.5-fold) and (~2.0-fold), respectively, compared to the wild-type strain in the presence of rice macerate. Although the complemented strain was able to restore the deficiency of the *xocR* mutant in cell motility (Figure 3), suggesting the construction of complemented strain is corrected. However, we always found the transcriptional level of both tested genes (*recN* and *trpE*) was highly similar with their cases in the *xocR* mutant under all testing conditions (data not shown). It is possible that the transcriptional level of both genes in the pBBR1-MCS5 vector was relatively high compared to their native levels in the genome, further suggesting the transcriptional regulation of *xocR* on both genes was probably precise in *Xoc*. Nevertheless, our results collectively suggest that the rice macerate may activate the positive regulation effect of *xocR* on the transcription of two known virulence-associated genes in *Xoc*. It is currently not known whether XocR directly binds the promoter region of *recN* or *trpE* to regulate their transcription in the presence of rice macerate or whether this regulation is indirect. RecN is a highly conserved, SMC (structural maintenance of chromosomes)-like protein in bacteria (Zeigler, 2003). It plays an important role in the repair of DNA double-strand breaks and therefore acts as a critical factor in maintaining genome integrity (Grove et al., 2009). Moreover, RecN also functions as a key component of the SOS response in different bacterial species, including *Haemophilus inlfuenzae, Bacillus subtilis* and *Pseudomonas fluorescens* (Alonso et al., 1993; Sweetman et al., 2005; Sanchez et al., 2006; Jin et al., 2007). However, the involvement of *recN* in the virulence of plant pathogenic bacteria is largely unknown. TrpE is one component of microbial anthranilate synthase (AS), which is a member of tryptophan biosynthetic pathway. AS catalyzes the formation of anthranilate from chorismate and ammonia (or glutamine), which is the first committed step branching from shikimate pathway toward the biosynthesis of l-tryptophan (Morollo and Eck, 2001). TrpE is able to catalyze the synthesis of anthranilate with ammonia as the source of nitrogen atom independently (Bauerle et al., 1987). The involvement of *trpE* in the virulence of plant pathogenic bacteria is also poorly understood.

## Concluding remarks

This study reports for the first time the presence of a PAB LuxR solo designated as XocR in *Xoc*. XocR shares high sequence similarity at amino-acid level with OryR from *Xoo*. XocR, responds to rice-derived unidentified compound(s) and regulates virulence on host rice and cell motility similar to OryR. We further provided evidence that XocR mediated *Xoc* virulence through the control of the transcription of two known virulence-associated genes (*recN* or *trpE*) in the presence of rice signal(s). Future studies will focus on the identification of the rice signal(s) that interacts with XocR, and the regulatory mechanisms of XocR on cell motility as well as the transcription of *recN* or *trpE* in *Xoc*.

### Conflict of interest statement

The authors declare that the research was conducted in the absence of any commercial or financial relationships that could be construed as a potential conflict of interest.
