# Supplementary material for: XocR, a LuxR solo required for virulence in Xanthomonas oryzae pv. oryzicola
Source: Front Cell Infect Microbiol. 2015 Apr 16;5:37. doi: 10.3389/fcimb.2015.00037 (PMC4399327; doi:10.3389/fcimb.2015.00037)
Supplement: Supplementary file 1 [file Table1.DOC]

**Table S1 Bacterial strains and plasmids used in this study**

| **Strains and plasmids** | | **Properties or characteristics ^a^** | **Source or reference** |
| --- | --- | --- | --- |
| Strains | | | |
| *Xanthomonas oryzae* pv. *oryzicola* | | | |
| Rs105 | | Wild-type strain Rs105, Rif^r^ | Lab collection |
| **Δ***xocR* | | *xocR* in-frame deletion mutant of strain Rs105, Rif^r^ | This study |
| Δ*xocR*(*xocR*) | | Mutant Δ*xocR* harboring plasmid pBBR- *xocR*, Rif^R^, Gm^R^ | This study |
| *Escherichia coli* | | | |
| DH5a | | F^-^, φ80d*lacZ*∆M15, ∆(*lacZYA*-*argF*)U169, *deoR*, *recA1*, *endA1*, *hsdR*17(r_k_^-^,m_k_^+^), *phoA*, *supE*44, λ^-^, *thi*-1, *gyrA*96 | Lab collection |
| Plasmids | | | |
| pMD19-T | Amp^R^ , ColE1 origin, T Simple vector | | TaKaRa |
| pK18mob*sacB* | Km^R^, oriT(RP4), *sacB*, *lacZ*alpha, Plac, Pmbi, Mobilization and counter selection | | [Schafer et al., 1994](#_ENREF_35) |
| pK18- *xocR* | Km^R^, pK18mob*sacB* with two *xocR* flanking fragments | | This study |
| pBBR1-MCS5 | Broad host range cloning vector, *lacZ*, Gm^R^ | | [Kovach et al., 1995](#_ENREF_23) |
| pBBR- *xocR* | Gm^R^ , pBBR1-*MCS5* with 1.66-kb fragment including *xocR* | | This study |

^a^ Rif^r^, Km^R^, Gm^R^ and Amp^R^ indicate resistance to rifamycin, kanamycin, gentamicin and ampicillin, respectively.
