# Supplementary material for: XocR, a LuxR solo required for virulence in Xanthomonas oryzae pv. oryzicola
Source: Front Cell Infect Microbiol. 2015 Apr 16;5:37. doi: 10.3389/fcimb.2015.00037 (PMC4399327; doi:10.3389/fcimb.2015.00037)
Supplement: Supplementary file 2 [file Table2.DOC]

**Table S2 Primers used for gene deletion and complementation in this study**

| **Primer** | **Sequence^a^** | **Purpose** |
| --- | --- | --- |
| *xocR*-1F | CGGGATCCCCGACCACATACAGCAGCGT (*Bam*HI) | To amplify a 627-bp upstream homologue arm of *XOC_1422* |
| *xocR*-1R | CCCAAGCTTAGCCATTGAGCGTTTGCGAC (*Hin*dIII) |
| *xocR*-2F | CCCAAGCTTAAGCGGTGGTGCGTGGTAT (*Hin*dIII) | To amplify a 583-bp downstream homologue arm of *XOC_1422* |
| *xocR*-2R | GCTCTAGAGGATCGGCATTGGGCAGAT (*Xba*I) |
| *xocR*H-F | CGGGATCCACACCACCGCCACCGACAAT (*Bam*HI) | To amplify a 1664-bp fragment containing intact  *xocR*  and its predicted promoter |
| *xocR*H-R | GCTCTAGATCCTCCGCCATGTCAAACCC (*Xba*I) |

^a^ Restriction digestion enzyme site was underlined.
