# Supplementary material for: XocR, a LuxR solo required for virulence in Xanthomonas oryzae pv. oryzicola
Source: Front Cell Infect Microbiol. 2015 Apr 16;5:37. doi: 10.3389/fcimb.2015.00037 (PMC4399327; doi:10.3389/fcimb.2015.00037)
Supplement: Supplementary file 3 [file Table3.DOC]

| **Gene fragment** | **Primer sequence for 5' sequence** | **Primer sequence for 3' sequence** | **Primer Source** |
| --- | --- | --- | --- |
| 16S rRNA | 5′-AATGGGCGCAAGCCTGATC-3′ | 5′-AACCACCACCTACGCACGC-3′ | [Qian et al., 2013b](#_ENREF_32) |
| *xoc_1737* | 5′-CAGCTGATGCTCCAGAAAGC-3′ | 5′-ACGCCTATGCCCGTAACAG-3′ | This study |
| *xoc_4211* | 5′-TACATGTACGGCGAGGGATT-3′ | 5′-CGACATCTTCCAGGTGGTG-3′ | This study |

**Table S3 Primers used in this study for quantitative real-time polymerase chain reaction**
