# Supplementary material for: XocR, a LuxR solo required for virulence in Xanthomonas oryzae pv. oryzicola
Source: Front Cell Infect Microbiol. 2015 Apr 16;5:37. doi: 10.3389/fcimb.2015.00037 (PMC4399327; doi:10.3389/fcimb.2015.00037)
Supplement: Supplementary file 4 [file Table4.DOC]

**Table S4. 29 characterized virulence-associated genes of *Xanthomonas oryzae* pv. *oryzicola* strain Rs105**

| **Gene** | **Putative product** | **Reference or source** |
| --- | --- | --- |
| *XOC_3805* | General secretion pathway protein E (XpsE) |  |
| *XOC_1135* | ATP-dependent protease La |  |
| *XOC_1737* | DNA repair protein RecN |  |
| *XOC_4457* | HrpF |  |
| *XOC_4044* | Pyruvate dehydrogenase E1 component |  |
| *XOC_3672* | Thiamine-phosphate pyrophosphorylase (ThiE) |  |
| *XOC_0106* | Exodeoxyribonuclease V, gamma subunit (RecC) |  |
| *XOC_3794* | cAMP-regulatory protein |  |
| *XOC_3402* | Transposase |  |
| *XOC_3794* | General secretion pathway protein D (XpsD) |  |
| *XOC_4486* | Membrane protein, putative |  |
| *XOC_4211* | Anthranilate synthase component I (TrpE) |  |
| *XOC_1450* | Conserved hypothetical protein |  |
| *XOC_1282* | Dipeptidyl carboxypeptidase I |  |
| *XOC_0969* | Conserved hypothetical protein |  |
| *XOC_0319* | Activator of XA21-mediated immunity Ax21 |  |
| *XOC_1601* | Cysteine protease |  |
| *XOC_3806* | Protease |  |
| *XOC_2128* | Polygalacturonase |  |
| *XOC _1105* | Amidoophosphoribosyltransferase (PurF) |  |
| *XOC_3845* | Glucose-1-phosphate  thymidylyltransferase (RfbA) |  |
| *XOC_2208* | Threonine synthase (ThrC) |  |
| *XOC_2950* | Tryptophan synthase, alphasubunit (TrpA) |  |
| *XOC_3054* | Asparagine synthetase B, (AsnB) |  |
| *XOC_2265* | RpfC |  |
| *XOC_0957* | Oxidative stress transcriptional regulator (OxyR) |  |
| *XOC_3811* | Disulfide isomerase (DsbC) |  |
| *XOC_0678* | Glucans biosynthesis glucosyltransferase (OpgH) |  |

**Reference**

Guo, W., Cui, Y.P., Li, Y.R., Che, Y.Z., Yuan, L., Zou, L.F., Zou, H.S., and Chen, G.Y. (2012). Identification of seven *Xanthomonas oryzae* pv. *oryzicola* genes potentially involved in pathogenesis in rice. *Microbiology* 158**,** 505-518. doi: 10.1099/mic.0.050419-0.

He, Y.W., and Zhang, L.H. (2008). Quorum sensing and virulence regulation in *Xanthomonas campestris*. *FEMS Microbiol Rev* 32**,** 842-857. doi: 10.1111/j.1574-6976.2008.00120.x.

Jiang, B.L., Liu, J., Chen, L.F., Ge, Y.Y., Hang, X.H., He, Y.Q., Tang, D.J., Lu, G.T., and Tang, J.L. (2008). DsbB is required for the pathogenesis process of *Xanthomonas campestris* pv. *campestris*. *Mol Plant Microbe Interact* 21**,** 1036-1045. doi: 10.1094/MPMI-21-8-1036.

Qian, G., Liu, C., Wu, G., Yin, F., Zhao, Y., Zhou, Y., Zhang, Y., Song, Z., Fan, J., Hu, B., and Liu, F. (2013a). AsnB, regulated by diffusible signal factor and global regulator Clp, is involved in aspartate metabolism, resistance to oxidative stress and virulence in *Xanthomonas oryzae* pv. oryzicola. *Mol Plant Pathol* 14**,** 145-157. doi: 10.1111/j.1364-3703.2012.00838.x.

Qian, G., Zhou, Y., Zhao, Y., Song, Z., Wang, S., Fan, J., Hu, B., Venturi, V., and Liu, F. (2013b). Proteomic Analysis Reveals Novel Extracellular Virulence-Associated Proteins and Functions Regulated by the Diffusible Signal Factor (DSF) in *Xanthomonas oryzae* pv. *oryzicola.* *J Proteome Res* 12**,** 3327-3341. doi: 10.1021/pr4001543.

Qu, Q., Morizono, H., Shi, D., Tuchman, M., and Caldovic, L. (2007). A novel bifunctional N-acetylglutamate synthase-kinase from *Xanthomonas campestris* that is closely related to mammalian N-acetylglutamate synthase. *BMC Biochem* 8**,** 4. doi: 10.1186/1471-2091-8-4.

Toledo, M.A., Schneider, D.R., Azzoni, A.R., Favaro, M.T., Pelloso, A.C., Santos, C.A., Saraiva, A.M., and Souza, A.P. (2011). Characterization of an oxidative stress response regulator, homologous to *Escherichia coli* OxyR, from the phytopathogen *Xylella fastidiosa*. *Protein Expr Purif* 75**,** 204-210. doi: 10.1016/j.pep.2010.10.004.

Zou, H.S., Yuan, L., Guo, W., Li, Y.R., Che, Y.Z., Zou, L.F., and Chen, G.Y. (2011). Construction of a Tn5-tagged mutant library of *Xanthomonas oryzae* pv. *oryzicola* as an invaluable resource for functional genomics. *Curr Microbiol* 62**,** 908-916. doi: 10.1007/s00284-010-9804-1.
