# Supplementary material for: XocR, a LuxR solo required for virulence in Xanthomonas oryzae pv. oryzicola
Source: Front Cell Infect Microbiol. 2015 Apr 16;5:37. doi: 10.3389/fcimb.2015.00037 (PMC4399327; doi:10.3389/fcimb.2015.00037)
Supplement: Supplementary file 5 [file Table5.DOC]

**Table S5. Comparison of the flagellar genes between *Xanthomonas oryzae* pv. *oryzicola* BLS256 and *X. oryzae* pv. *oryzae* KACC 10331**

^a^ *Xanthomonas oryzae* pv. *oryzicola* BLS256, the accession number in NCBI (<http://www.ncbi.nlm.nih.gov/>) is NC_017267.

| ***Xoc***  **BLS256 ^a^** | ***Xoo***  **KACC 10331 ^b^** | **Identity** | **E-value** | **Putative product ^c^** |
| --- | --- | --- | --- | --- |
| *XOC_2390* | XOO2566 | 98% | 3e-051 | flagellar protein |
| *XOC_2386* | XOO2569 | 99% | 5e-070 | flagellar basal body rod protein FlgB |
| *XOC_2385* | XOO2570 | 99% | 3e-067 | flagellar basal body rod protein FlgC |
| *XOC_2384* | XOO2571 | 98% | e-119 | flagellar basal body rod modification protein |
| *XOC_2382* | XOO2572 | 99% | 0.0 | flagellar hook protein FlgE |
| *XOC_2381* | XOO2574 | 98% | e-139 | flagellar basal body rod protein FlgF |
| *XOC_2380* | XOO2575 | 100% | e-147 | flagellar basal body rod protein FlgG |
| *XOC_2379* | XOO2576 | 99% | e-127 | flagellar basal body L-ring protein |
| *XOC_2378* | XOO2577 | 99% | 0.0 | flagellar basal body P-ring protein |
| *XOC_2377* | XOO2578 | 99% | 0.0 | flagellar rod assembly protein FlgJ |
| *XOC_2376* | XOO2579 | 99% | 0.0 | flagellar hook-associated protein FlgK |
| *XOC_2375* | XOO2580 | 99% | 0.0 | flagellar hook-associated protein FlgL |
| *XOC_2374* | XOO2581 | 98% | 0.0 | flagellin |
| *XOC_2373* | XOO2582 | 99% | 0.0 | flagellar protein |
| *XOC_2372* | XOO2583 | 98% | 1e-073 | flagellar protein |
| *XOC_2352* | XOO2601 | 98% | 0.0 | flagellar MS-ring protein |
| *XOC _2351* | XOO2602 | 99% | e-178 | flagellar protein |
| *XOC_2349* | XOO2604 | 99% | 0.0 | flagellar protein |
| *XOC_2348* | XOO2605 | 98% | 5e-077 | flagellar FliJ protein |
| *XOC_2347* | XOO2606 | 96% | 0.0 | flagellar protein |
| *XOC_2346* | XOO2607 | 97% | 3e-094 | flagellar protein |
| *XOC_2345* | XOO2608 | 99% | 0.0 | flagellar motor switch protein FliM |
| *XOC_2344* | XOO2609 | 99% | 2e-058 | flagellar protein |
| *XOC_2343* | XOO2610 | 95% | 1e-068 | flagellar protein |
| *XOC_2342* | XOO2611 | 98% | e-155 | flagellar biosynthesis protein FliP |
| *XOC_2333* | XOO2618 | 99% | 0.0 | flagellar biosynthesis protein FlhA |
| *XOC_2331* | XOO2619 | 95% | 0.0 | flagellar biosynthesis regulator FlhF |
| *XOC_2330* | XOO2620 | 100% | e-165 | flagellar biosynthesis switch protein |

^b^ *Xanthomonas. oryzae* pv. *oryzae* KACC 10331, the accession number in NCBI (<http://www.ncbi.nlm.nih.gov/>) is NC_006834.

^c^ The flagellar genes are positively regulated by OryR by two-fold or more. OryR is a PAB LuxR solo from *Xoo* .
