# Supplementary material for: XocR, a LuxR solo required for virulence in Xanthomonas oryzae pv. oryzicola
Source: Front Cell Infect Microbiol. 2015 Apr 16;5:37. doi: 10.3389/fcimb.2015.00037 (PMC4399327; doi:10.3389/fcimb.2015.00037)
Supplement: Supplementary file 6 [file Presentation1.PDF]

Supplementary file

XocR, a LuxR solo required for virulence in *Xanthomonas oryzae* pv. *oryzicola*

Huiyong Xu[b$], Yancun Zhao[a$], Guoliang Qian[b], and Fengquan Liu[a, b*]

[a] Institute of Plant Protection, Jiangsu Academy of Agricultural Science, Nanjing 210014, P.R. China

[b]College of Plant Protection, Nanjing Agricultural University, Nanjing 210095, China/Key Laboratory of Integrated Management of Crop Diseases and Pests (Nanjing Agricultural University), Ministry of Education

[c]International centre for Genetic engineering and biotechnology, Padriciano 99, 34149, Trieste, Italy

[*]To whom correspondence should be addressed. Tel: +86-25-84396726. Fax: +86-25-84395325. E-mail: fqliu20011@sina.com

$The first two authors contribute equally to this work.

Results and Figures

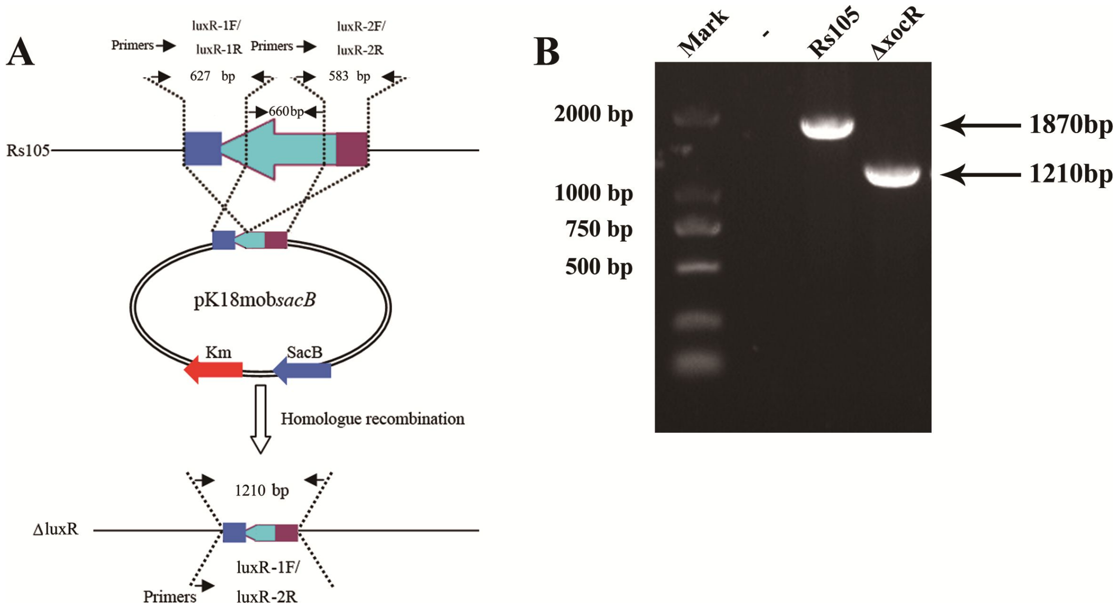

Figure S1. The scheme of the *xocR* mutant construction and molecular confirmation in *Xanthomonas oryzae* pv. *oryzicola*. (A) The gene deletion scheme is shown. The 627-bp (amplified by *xocR*-1F/ *xocR*-1R) and 583-bp (amplified by *xocR*-2F/ *xocR*-2R) DNA fragments were used as 5' and 3' fragments for homologue recombination, respectively. The internal 660-bp DNA fragment was deleted in the *xocR* mutant. The *xocR*-1F/ *xocR*-2R primer was used for molecular confirmation of the *xocR* mutant. If the 660-bp internal fragment of *xocR* was successfully deleted, a 1210-bp DNA fragment would be amplified from the *xocR* mutant. (B) Polymerase chain reaction (PCR) confirmation of the *xocR* mutant is shown. Due to deletion of the 660-bp internal fragment of *xocR*, only a ~1.3-kb DNA fragment was amplified from the *xocR* -deletion mutant, which was consistent with the expected result described in part A.

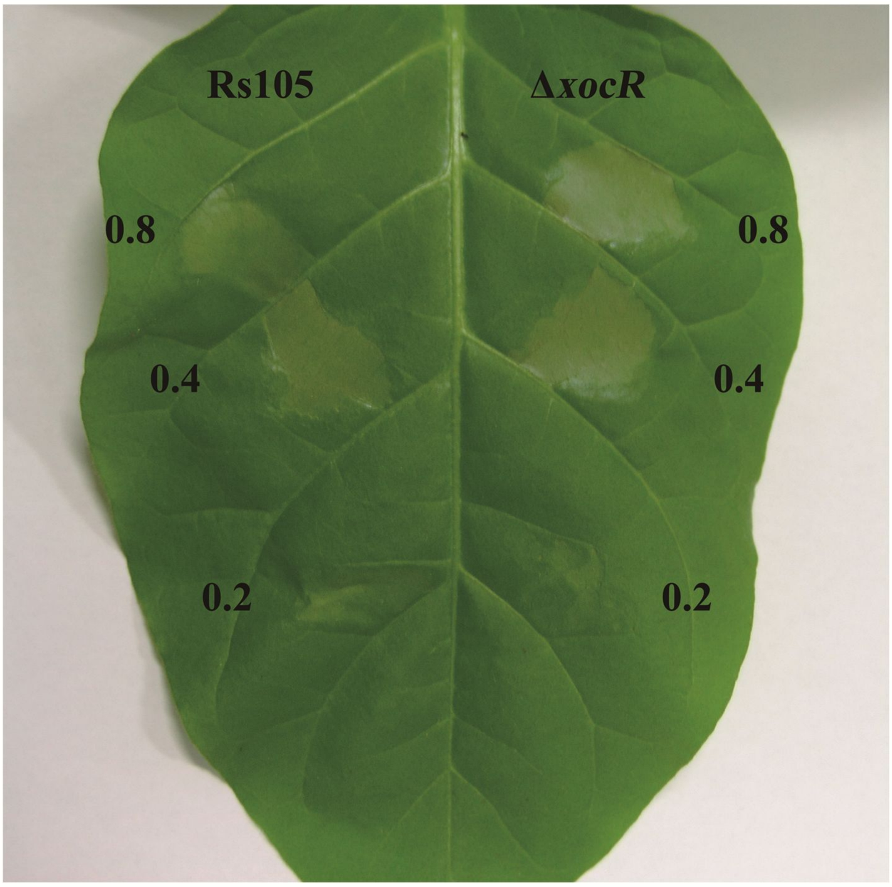

Figure S2. Hypersensitive response assay of *Xanthomonas. oryzae* pv. *oryzicola* in tobacco by using differentially inoculated cell density. Phenotypes for the hypersensitive response (HR) that is a programmed cell death on non-host tobacco by inoculation with *X. oryzae* pv. *oryzicola* strains. Rs105, wild-type strain of *X. oryzae* pv. *oryzicola*; Δ*xocR*, *xocR* gene deletion mutant of *X. oryzae* pv. *oryzicola*. The numbers in the figure represent the cell density that is expressed by optical density at 600 nm ($OD600_{nm}$).

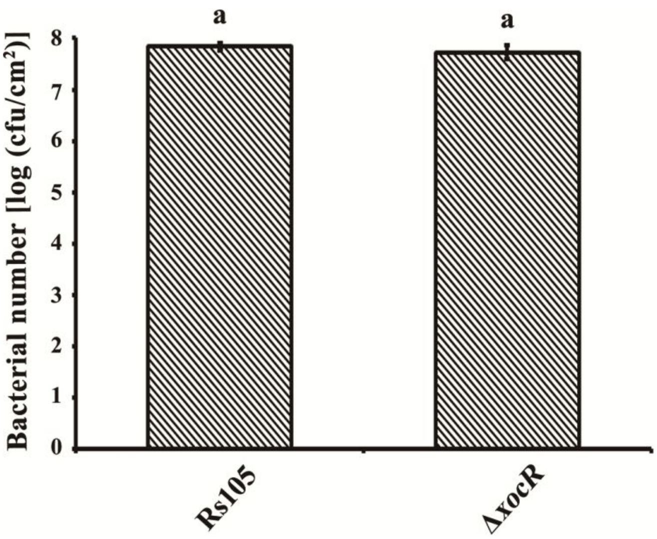

Figure S3. Determination of the growth ability of the *xocR* deletion mutant of *Xanthomonas oryzae* pv. *oryzicola in planta.* Bacteria were recovered from infected rice leaves (cv. Shanyou63) 7 days after inoculation. Cfu is the abbreviation of Colony-Forming Units. Three replicates for each treatment were used, and the experiment was performed three times. Vertical bars represent standard errors. Identical letters above the data bars indicate that there is no significant difference between the wild-type and the *xocR* mutant (P>0.05; t test).

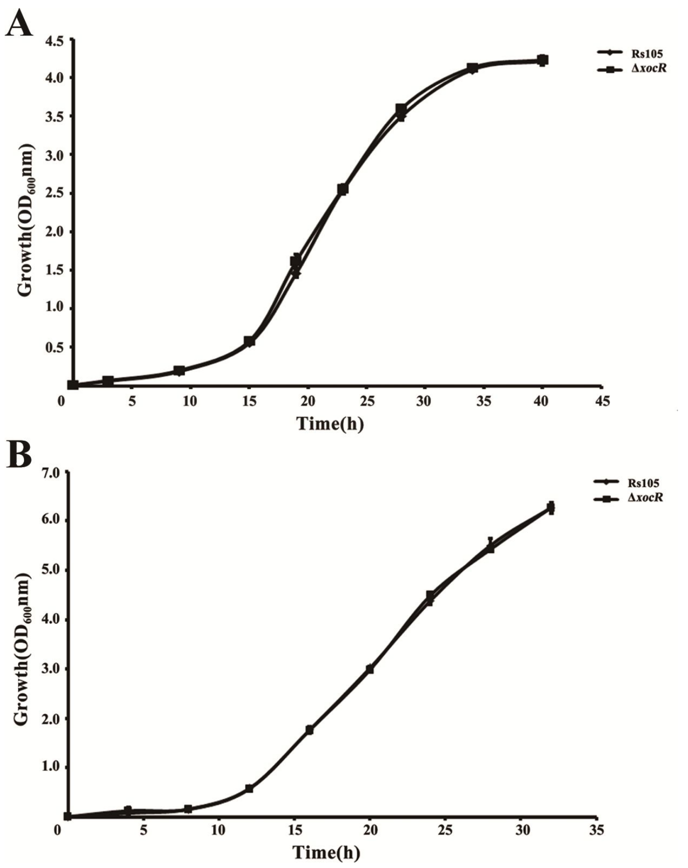

Figure S4. Determination of the growth ability of the *xocR* deletion mutant of *Xanthomonas oryzae* pv. *oryzicola* in media. (A) Growth rate of wild-type strain and the *xocR* mutant in nutrient-rich NB broth; (B) Growth rate of wild-type strain and the *xocR* mutant in NB broth supplemented with rice macerate. The *xocR* mutant displayed wild-type growth level both in these two media. Rs105, wild-type strain of *X. oryzae* pv. *oryzicola*; Δ*xocR*, *xocR* gene deletion mutant of *X. oryzae* pv. *oryzicola*. Three replicates were used for each treatment, and the experiment was repeated three times. Vertical bars represent standard errors.

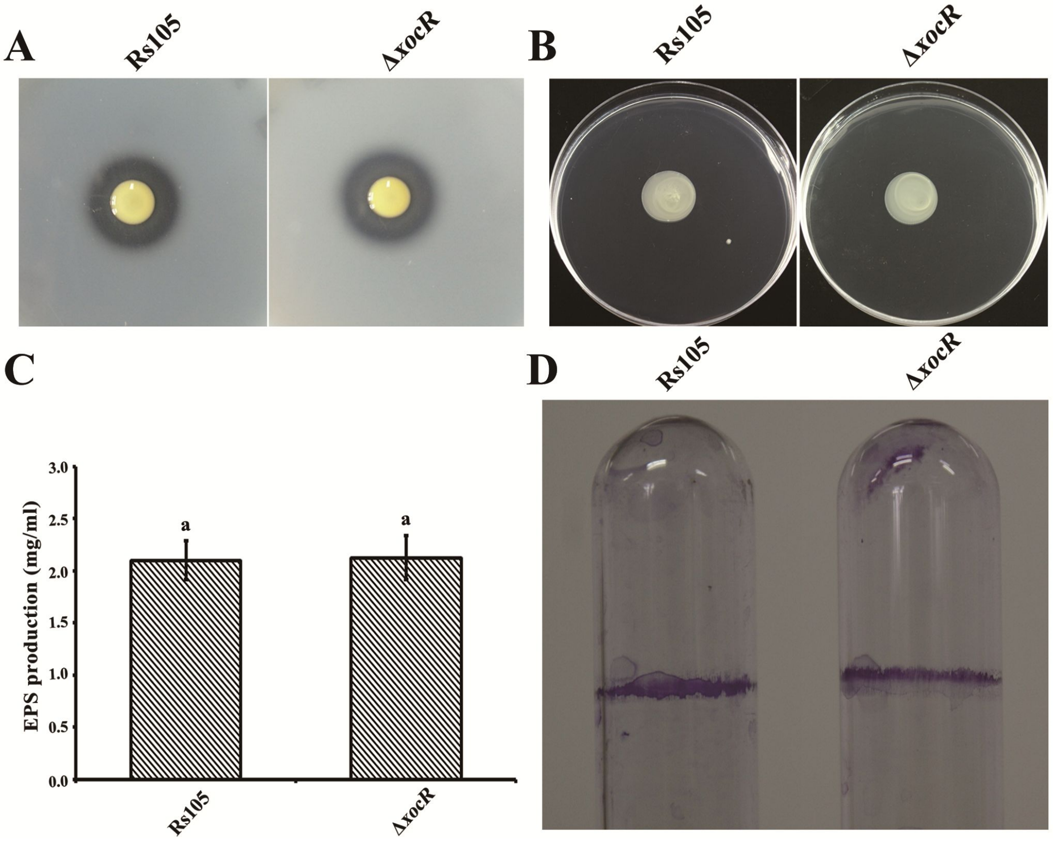

Figure S5. Effect of the *xocR* mutation on *Xanthomonas oryzae* pv. *oryzicola* protease activity (A), motility (B), extracellular polysaccharide production (C) and biofilms (D) in nutrient-rich broth (NB) without rice macerate. No visible difference between wild-type strain the *xocR* deletion mutant was observed under the tested condition. Rs105, wild-type strain of *X. oryzae* pv. *oryzicola*; Δ*xocR*, *xocR* -deletion mutant of *X. oryzae* pv. *oryzicola.* Three replicates were used for each treatment, and the experiment was performed three times. Vertical bars represent standard errors. Identical letters above the data bars indicate that there is no significant difference between the wild-type and the *xocR* mutant (P>0.05; *t* test).

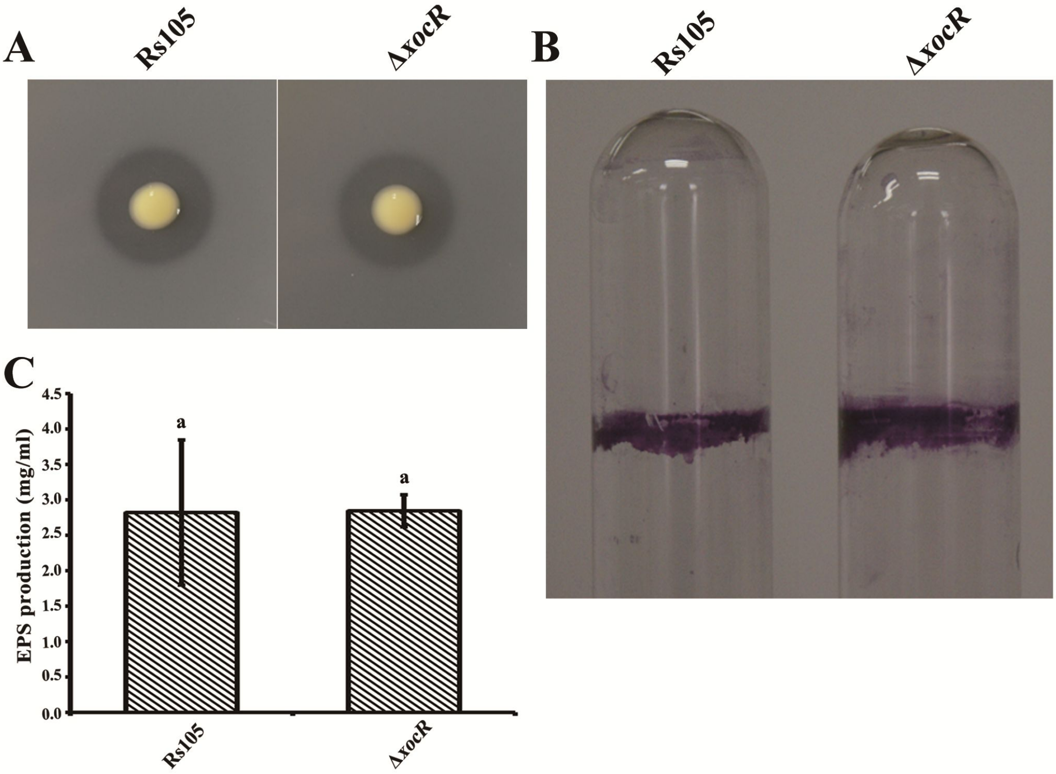

Figure S6. Effect of the *xocR* mutation on *Xanthomonas oryzae* pv. *oryzicola* protease activity (A), biofilms (B) and extracellular polysaccharide production (C) in nutrient broth (NB) with rice macerate. No visible difference between wild-type strain the *xocR* deletion mutant was observed under the tested condition. Rs105, wild-type strain of *X. oryzae* pv. *oryzicola*; Δ*xocR*, *xocR* -deletion mutant of *X. oryzae* pv. *oryzicola.* Three replicates were used for each treatment, and the experiment was performed three times. Vertical bars represent standard errors. Identical letters above the data bars indicate that there is no significant difference between the wild-type and the *xocR* mutant (P>0.05; t test).
